# The complete mitochondrial genome of *Greenidea ficicola* (Hemiptera: Aphididae: Greenideinae), a pest of *Ficus*

**DOI:** 10.1080/23802359.2019.1699472

**Published:** 2019-12-12

**Authors:** Qian Liu, Hui Zhang, Jun Deng, Xiaolan Lin, Xiaolei Huang

**Affiliations:** State Key Laboratory of Ecological Pest Control for Fujian and Taiwan Crops, College of Plant Protection, Fujian Agriculture and Forestry University, Fuzhou, China

**Keywords:** Mitogenome, repeat region, control region, phylogeny

## Abstract

In this study, the complete mitochondrial genome of the pest aphid *Greenidea ficicola* was determined. The mitogenome was 17,361 bp in length, containing 13 protein-coding genes, 22 tRNA genes, 2 rRNA genes, 1 long control region located between *srRNA* and *tRNA^Ile^*, and a repeat region located between *tRNA^Glu^* and *tRNA^Phe^*. Thirteen protein-coding genes have typical ATN start codon and TAA termination codon. All tRNAs were predicted to contain typical clover-leaf secondary structures except *tRNA^Ser(gct)^.* The length of *lrRNA* and *srRNA* are 1270 bp and 773 bp, respectively. Phylogenetic analysis shows that Greenideinae speices form a highly supported clade.

*Greenidea ficicola* (Greenideinae: Greenideini) mainly feeds on *Ficus* plants, such as *Ficus microcarpa*, *F. altissima*, *F. benjamina*, on the undersides of young leaves and on the shoots. It sometimes concentrated on plants of other families, such as *Litchi chinensis*, *Psidium guajava*, *Glycosmis pentaphylla*. *Greenidea ficicola* is widely distributed in Asia (Blackman and Eastop 2000) and has been introduced to many other areas, such as Tunisia, Malta, Italy, and Hawaii (USA). To date, in Greenideinae, two complete mitochondrial genomes of *Greenidea psidii* and *Cervaphis quercus* have been reported (Wang et al. 2014; Chen et al. 2019).

Samples of *G. ficicola* were collected in February 2017 from Guangzhou City, Guangdong province, China. Voucher specimen (HL20170210-1) was deposited in the Insect Systematics and Diversity Lab at Fujian Agriculture and Forestry University, Fuzhou, China. The mitogenome was sequenced on an Illumina platform and assembled using NovoPlasty v. 2.7.1 (Dierckxsens et al. 2017). Then, the MITOS Webserver (Bernt et al. 2013) was used to annotate the mitogenome.

The total length of *G. ficicola* mitogenome is 17,361 bp (GenBank accession number: MN704283), longer than majority of published aphid mitogenomes. The overall base composition was A (46.9%), T (38.8%), C (9.0%), and G (5.3%), with a strong bias toward A + T (85.7%). The mitogenome contains 13 protein-coding genes (PCGs), 22 tRNA genes (tRNAs), 2 rRNA genes (rRNAs), and 1 control region. Gene order was conserved and it was identical to the inferred ancestral arrangement of insects (Clary and Wolstenholme 1985) and to that of *G. psidii* (Chen et al. 2019). Fourteen tRNAs and nine PCGs are transcribed on the forward strand (J-strand), the remaining genes being oriented on the reverse strand (N-strand). All PCGs initiate with typical ATN as the start codon (six ATA, five ATT, and two ATG) and terminate with conventional stop codon TAA. The mean length of tRNAs is 67 bp, ranging from 62 bp to 73 bp. All tRNAs exhibit the typical clover-leaf like secondary structures except *tRNA^Ser(gct)^*. The length of *lrRNA* and *srRNA* are 1270 bp and 773 bp, with 85.2% and 84.6% A + T content, respectively. The control region (1598 bp) with a high A + T content (92.6%) is longer than that of most other aphid species, it is located between *srRNA* and *tRNA^Ile^* and contains a long tandem repeats (861 bp). In addition, a long repeat region (950 bp) between *tRNA^Glu^* and *tRNA^Phe^* exists in the *G. ficicola* mitogenome, which was thought to be highly species-specific and unique to some Hemiptera lineages (Wang et al. 2013, 2015).

Sequences of all 13 PCGs were extracted from the mitochondrial genomes of *G. ficicola* and 33 other aphids, including one outgroup species *Adelges laricis*. A maximum-likelihood phylogenetic tree (Figure 1) was reconstructed using IQ-TREE (Nguyen et al. 2015), which showed that *G. ficicola* and other two Greenideinae species clustered together with strong support, and other subfamilies also formed separate clades except Eriosomatinae. Although with a low support, our phylogenetic tree indicated that two Erisomatini species grouped with Greenideinae, which is also reported in other studies (Nováková et al. 2013; Lee et al. 2019).

**Figure 1.**
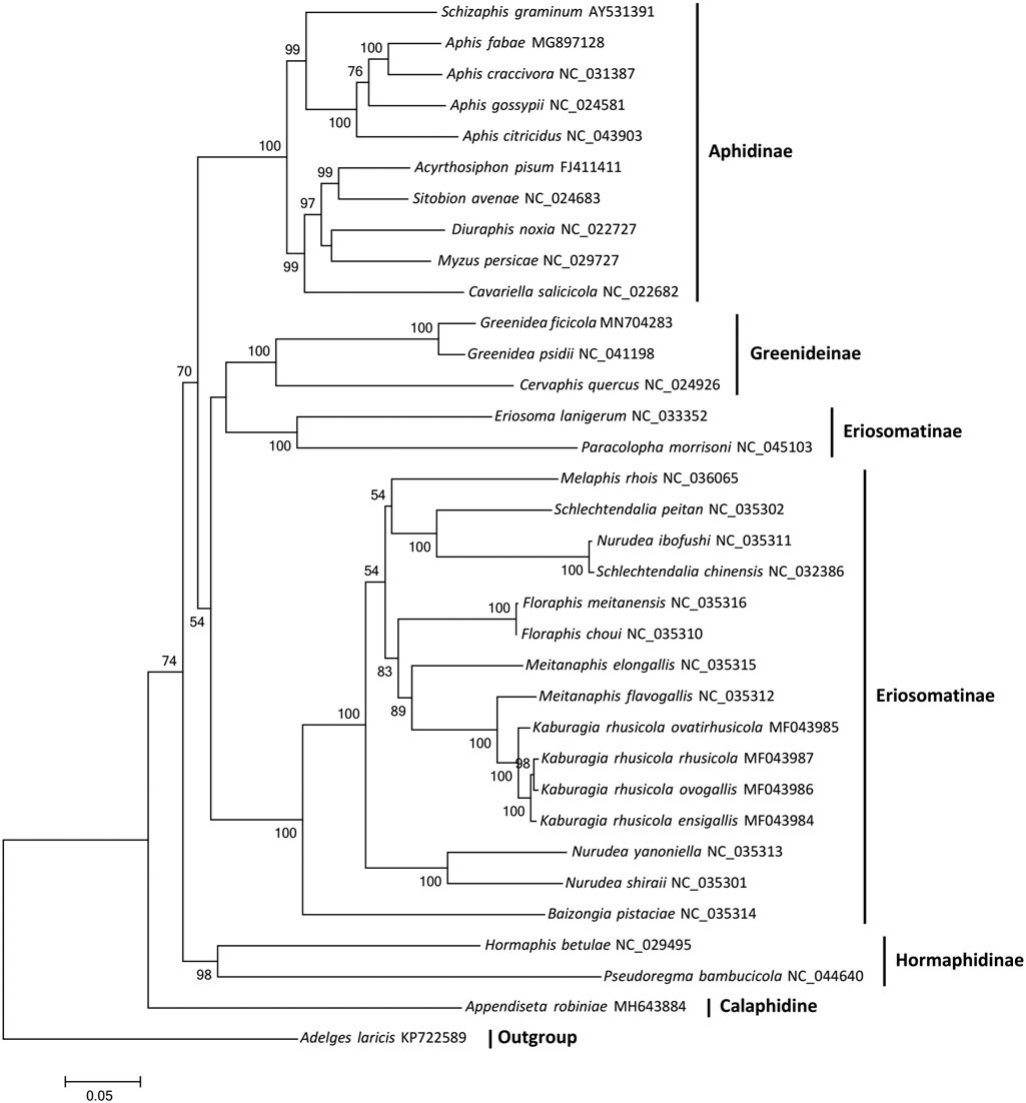
The maximum-likelihood tree of *G. ficicola* and 33 other aphids based on sequences of 13 protein-coding genes. Numbers above the branches indicate the bootstrap support values, and values lower than 50 are not shown.
